# Caste-specific development of the dopaminergic system during metamorphosis in female honey bees

**DOI:** 10.1371/journal.pone.0206624

**Published:** 2018-10-29

**Authors:** Ken Sasaki, Atsushi Ugajin, Ken-ichi Harano

**Affiliations:** Graduate School of Agriculture, Tamagawa University, Machida, Tokyo, Japan; USDA Agricultural Research Service, UNITED STATES

## Abstract

Caste-specific differences in the dopaminergic systems of social insects assist in maintaining caste-specific behavior. To determine how caste differences in the honey bee occur during metamorphosis, a number of comparative analyses between castes were performed including comprehensive quantification of: levels of dopamine and its metabolite in the brain, the gene expression levels of enzymes involved in dopamine biosynthesis and conversion as well as expression levels of dopamine receptors and a dopamine transporter. Dopamine levels standardized to the protein contents of a whole brain at the day of eclosion in queens were 3.6-fold higher than those in workers. Dopamine levels increased until eclosion (7 days) in queens, whereas those in workers increased until 5–6 days before eclosion and then maintained until eclosion (10 days). These caste-specific dopamine dynamics in the brain were supported by the higher expression of genes (*Amddc* and *Amth*) encoding enzymes involved in dopamine synthesis in queens. The distribution of cells expressing *Amddc* in the brain revealed that soma clusters of dopaminergic cells were similar between castes at 7–8 days after pupation, suggesting the upregulation of *Amddc* expression in some cells in queens rather than addition of cell clusters. In contrast, genes encoding dopamine receptors were downregulated in queens or showed similar expression levels between castes. The expression of genes encoding dopamine transporters did not differ between castes. These results reveal the developmental process of caste-specific dopaminergic systems during metamorphosis in the honey bee, suggesting caste-specific behavior and division of reproduction in this highly eusocial species.

## Introduction

Polyphenism is a form of phenotypic plasticity involving discontinuous morphological and physiological expression seen widely in animals. This occurs through different developmental processes for adaptation to different environments, such as temperature-dependent sex differentiation in reptiles and fish [1–3], alternative migratory tactics in salmonids [4], density-dependent phases in locusts [5,6], and seasonal polymorphism in butterflies [7,8]. Caste differentiation in social insects is also a polyphenism generated in response to different nutritional states during the larval stage [9,10]. In the honey bee, some female larvae fed royal jelly develop into reproductive individuals (queens), whereas others fed limited royal jelly subsidized with honey and pollen develop into nonreproductive individuals (workers). The detection of nutritional status involves activation of diverse genes including those encoding insulin, target of rapamycin (TOR) and epidermal growth factor (EGF) activated by royalactin in royal jelly [11]. These signaling pathways enhance the hemolymph levels of juvenile hormone resulting in the development of queens [10]. At the end of metamorphic adult development, the dimorphism of the external and internal morphologies between castes results in specialized organs that function division of honey bee labor.

The caste-specific morphology and physiology in adults maintains the caste-specific behavior and division of reproduction. Various morphological differences of the central nervous system including the structure of brain subcompartments (antennal lobes and mushroom bodies) [12–14] and the terminal abdominal ganglion [15,16] form during metamorphosis and differentiate the castes until adult emergence. Therefore, clarification of the caste-specific developmental processes of the brain during metamorphosis might help us to understand the origin of the division of labor in social insects.

One caste-specific physiological character in the adult honey bee brain is the dopaminergic system [17,18]. Dopamine functions as a neuroactive substance that transmits neural signals (neurotransmitter), modulates neuronal activities in local neural circuits (neuromodulator), and causes the expression of certain genes in target cells (neurohormone) in insects [19–24]. Dopamine can promote female reproduction including ovarian development, mating receptivity and oviposition in solitary [25–27] and eusocial species [27–30]. In the honey bee, virgin queens have an approximately four-fold higher level of dopamine in the brain compared with normal workers [18]. These higher dopamine levels in virgin queens promote queen-specific behaviors, including high locomotor activity [31] and high aggressiveness towards other virgin queens [32]. In reproductive workers, the brain levels of dopamine are positively correlated with ovarian activities [33,34], and dopamine can activate ovarian development under queenless conditions [35]. Thus, the higher dopamine levels in reproductive honey bee females are associated with reproductive states and behaviors. Given that the dopamine levels in queens are already high just before emergence [31], it is expected that the dopaminergic caste differences in the brain are formed during metamorphosis. However, to our knowledge, no published studies have yet compared the brain dopaminergic systems between honey bee castes during metamorphosis.

There are several reports on the dynamics of dopamine levels [36,37] and expression of dopamine receptors [37–39] in the brain during metamorphosis of honey bee workers. In workers, brain levels of dopamine change with age and peak during the pupal stage [36,37]. The expression of dopamine receptors fluctuates depending on pupal age and receptor subtype, but is not directly correlated with levels of dopamine in the brain [37]. The spatial distributions of cells expressing each dopamine receptor gene in the brain, especially in the mushroom bodies, do not completely overlap, suggesting distinct roles for each receptor [38,39]. Thus, the dopaminergic system in honey bee workers is an important model for ligand–receptor relationships in insect brains. However, those reports only described this system in worker brains, and little is known about the development of the dopaminergic system in queen honey bees and how caste differences appear.

The present study focuses on caste differences in dopaminergic systems in the pupal brain of the honey bee. Developmental processes for dopamine biosynthesis, dopamine signaling, and dopamine conversion or uptake are considered to be important to understand caste-specific dopaminergic systems. Therefore, we compared the levels of dopamine and its metabolite in the brain, as well as the gene expression levels of enzymes involved in dopamine biosynthesis and conversion, dopamine receptors, and a dopamine transporter between castes, and determined how the caste-specific dopaminergic systems in each adult brain are generated.

## Materials and methods

### Collection of pupae and adults

Two queenright colonies of the European honey bee (*Apis mellifera*) were used to collect pupae and adult queens and workers. Queen pupae and adults were reared by a standard queen-rearing method with queenright colonies [40]. Briefly, newly hatched larvae from the source queenright colonies were placed into plastic queen cups and transferred to queenright breeding colonies with two-storied hives. The larvae grafted into queen cups were placed in a queenless area created by a horizontal queen excluder in the queenright colonies. Since pupal periods of female honey bees are shorter in queens (7 days) than in workers (10 days) [13], time points for collection of pupae were designed as 0–1, 2–3, and 4–5 days after pupation (6–7, 4–5, and 2–3 days before eclosion) for queens, and 0–1, 2–3, 4–5, and 7–8 days after pupation (9–10, 7–8, 5–6, and 2–3 days before eclosion) for workers, respectively. We initially compared the data at the same day ages after pupation (0–1, 2–3 and 4–5 days) between castes, but data on the days before eclosion fitted between castes more accurately. Therefore, we adopted the days before eclosion for comparisons, although these were slightly different between castes. We also collected and brought the sealed queen cells to the laboratory 2 days before emergence and kept them in individual plastic containers (60 mm height × 45 mm i.d.) at 34°C and then harvested any newly emerged adults (7 days after pupation). Worker pupae were collected from the capped worker comb cells in the same source queenright colonies. Newly emerged adult workers (10 days after pupation) were also collected from the worker comb cells and transferred into an incubator at 34°C. All ages of pupae that we collected and newly emerged adults of both castes were frozen with liquid nitrogen and stored until used for measurements of biogenic amines and gene expression. Several newly emerged adult queens (7 days after pupation) and 7–8-day-old worker pupae were not frozen with liquid nitrogen and used for *in situ* hybridization. In all samples, we collected the pupae and newly emerged adults on the basis of the days after pupation. We also confirmed the pupal ages by the degree of pigmentation on the basis of [13,41]. There was no inconsistency between the pupal ages that we collected and the ages estimated by the pupal pigmentation.

### Measurements of dopamine and its metabolite in the brain

Frozen brains of pupae or adults were dissected in ice-cold honey bee saline (128.33 mM NaCl, 2.68 mM KCl, 1.80 mM CaCl_2_, pH 6.7) on a Peltier cooling unit (Kenis Ltd, Osaka, Japan) at approximately 4°C under a microscope. Dissected brains with a subesophageal zone were homogenized with a microglass homogenizer in 50 μL ice-cold 0.1 M perchloric acid containing 0.1 ng/μL 3,4-dihydroxyphenylacetic acid for 2 min. Each sample was then transferred into a 1.5-mL microcentrifuge tube and centrifuged at 15,000 *g* for 30 min at 4°C. Supernatants were transferred into microvials for analysis by high-performance liquid chromatography with electrochemical detection (HPLC-ECD).

A HPLC-ECD method developed by [42] was used to analyze the levels of dopamine and *N*-acetyldopamine (NADA, a dopamine metabolite). The HPLC system comprised a solvent delivery pump (PU-2080, JASCO, Tokyo, Japan), a refrigerated automatic injector (AS-2057, JASCO), and a C18 reversed-phase column (250 mm × 4.6 mm id., 5-μm average particle size, UG 120, Shiseido, Tokyo, Japan) maintained at 35°C. An electrochemical detector (ECD-700, EICOM, Kyoto, Japan) set at 0.7 V was used under 35°C. The mobile phase contained 0.18 M monochloroacetic acid and 40 μM 2Na-EDTA, which was adjusted to pH 3.6 with NaOH. Into this solution, 1.62 mM sodium-1-octanesulfonate and 5% CH_3_CN were added. The flow rate was kept constant at 0.7 mL/min. External standards were run before and after the sample runs for the identification and quantification of dopamine and NADA. Each biogenic amine peak was identified by comparing both the retention time and hydrodynamic voltammograms with those of the standards. Measurements based on the peak area of the chromatograms were obtained by calculating the ratio of the peak area of a substance to the peak area of the standard.

To standardize the dopamine levels in the brains based on protein content, protein concentrations were quantified by the Bradford method [43]. A protein pellet precipitated after biogenic amine extraction was neutralized with 50 μL 0.5 M NaOH. After ultrasonic dissolution of the pellet for 15 min, the solution was diluted with 200 μL 0.1 M phosphate buffer (pH 7.0). As a standard solution, bovine serum albumin was dissolved in 0.5 M NaOH with 0.1 M phosphate buffer (1:4, 5mg/mL) and diluted with the same buffer 1/10, 1/20, 1/40, or 1/80 fold. The samples and the standard solution were reacted using a protein assay dye reagent (500–0006, Bio-Rad, Hercules, CA, USA) in a 96 well-plate and mixed for 5 min. The absorption was measured by a microplate reader (SH-1200Lab, Corona Electric, Ibaraki, Japan) with a 595-nm wave length. Protein concentrations in the brains were calculated based on a calibration curve of the standard solutions.

### Measurements of dopamine-related gene expression in the brain

Frozen brains of pupae or adults were dissected in ice-cold double-sterilized 0.1 M phosphate buffer (pH. 7.0) on a cooling unit at approximately 4°C under a dissecting microscope. Dissected brains, including the subesophageal zone, were homogenized with an electric homogenizer (T10+S10N-5G, IKA Works, Staufen, Germany) in extraction buffer from a NucleoSpin RNA isolation kit (Macherey-Nagel, Düren, Germany). Total RNA was extracted from two brains using the RNA isolation kit according to the manufacturer’s instructions. During RNA extraction, the RNA was treated with rDNase for 15 min to remove genomic DNA. The quality and quantity of extracted RNA were determined at 230, 260, and 280 nm using a microvolume spectrophotometer (Nanodrop^TM^ 2000, Thermo-Fisher Scientific, MA, USA). For single-strand cDNA synthesis, DNase-treated mRNA (500 ng) was transcribed using a high-capacity cDNA Reverse Transcription kit (Applied Biosystems, CA, USA) according to the manufacturer’s instructions. Negative control samples without the reverse transcriptase were also treated using the same procedure.

Three genes encoding enzymes involved in dopamine biosynthesis (tyrosine hydroxylase [*Amth*] and DOPA decarboxylase [*Amddc*]) or degradation (dopamine *N*-acetyltransferase [*Amnat*]), a dopamine transporter gene (*Amdat*) and four dopamine receptor genes (dopamine receptors 1, 2 and 3 [*Amdop1*, *Amdop2*, and *Amdop3*, respectively] and dopamine-ecdysteroid receptor [*Amgpcr19*]) were selected as targets for quantitative PCR (qPCR) (S1 Table). Four reference genes (actin [*Amact*], ribosomal protein subunit 49 [*Amrp49*], elongation factor 1 α1 [*Amef1a1*], and tbp-association factor [*Amtbpaf*]) were examined with sets of primers [44] (S1 Table). The primer sequences of target genes were designed using Primer 3 Plus (www.bioinformatics.nl/cgi-bin/primer3plus/primer3plus.cgi) (S1 Table), but those of *Amdop1* and *Amgpcr19* were applied from [45]. Standard curves were generated for each target and reference gene (1, 1/5, 1/10, and 1/100 dilutions). To make these standard curves, the qPCR template was cDNAs from newly emerged adult workers. The standard curves were based on the relative concentration of cDNA and the quantification cycle (Cq) required for each qPCR to cross a threshold fluorescent intensity within the linear portion of the amplification curve. The qPCR was performed using a KAPA SYBR FAST qPCR kit (KAPA biosystems, Nippon Genetics, Tokyo, Japan) with a real-time PCR system (Eco, Illumina, San Diego, CA, USA). Each reaction mixture (total volume 20 μL) comprised 10 μL of a KAPA SYBR Universal qPCR mix, 0.4 μL each of the forward and reverse primers (10 μM), 7.2 μL RNase-free water, and 2 μL of the cDNA template. The temperature profile for amplifying the target gene and reference gene fragments was 95°C for 1 min, followed by 40 cycles of 95°C for 3 s, and 60°C for 20 s. An individual sample was repeated twice in a single run of the qPCR. Although to take more technical replicates in an individual sample within a well-plate might result in less variation, the variations of average Cqs among biological replicates were significantly larger than those of differences between duplicate Cqs or not significantly different between them (S2 Table), suggesting small influences of technical replicates by duplicates. The amplification of the single product was confirmed by dissociation curve analysis using the real-time PCR system.

To estimate the mRNA expression levels of each dopamine-related gene, we recorded the Cq values of reference and target genes. The suitability of four reference genes as internal control genes (*Amact*, *Amrp49*, *Amef1a1*, and *Amtbpaf*) was evaluated using the software BestKeeper [46] and examined by a one-way ANOVA. The Cq values of *Amtbpaf* were the most stable in BestKeeper and were not significantly different among the developmental stages that we examined (ANOVA, d.f. = 6, F = 1.680, P = 0.163, N = 35, five samples each). Therefore, we normalized the expression levels of target genes by using expression levels of *Amtbpaf*. For quantification of the relative gene expression levels, the ΔΔCq method was adopted. For each sample, the ΔCq (target Cq–*Amtbpaf* Cq) were calculated, and then the ΔCq were normalized relative to an average ΔCq of control samples (adult workers) (ΔΔCq). The values of ΔΔCq were used for the calculation as 2^-ΔΔCq^. The ΔΔCq method requires an amplification efficiency of nearly 1.0 for both the reference gene and target genes. The amplification efficiencies were 1.052 in *Amtbpaf* and ranging from 1.018 to 1.128 in the target genes (S3 Table). These analyses were performed with reference to the Minimum Information for Publication of Quantitative Real-Time PCR Experiments (MIQE) guidelines [47].

### Localization of *Amddc* cells in the brains

To compare the soma clusters of dopaminergic cells in the brain between same-aged queens and workers, the brains of newly emerged queens (7 days after pupation) and worker pupae 2–3 days before eclosion (7–8 days after pupation) were stained by *in situ* hybridization using digoxigenin (DIG)-labeled riboprobes for *Amddc*. Although neuropiles may still be developing in the brains of worker pupae 7–8 days after pupation, the basic structure of the brain 6 days after pupation is that of an adult brain [13,48], suggesting it is comparable with the adult brain in queens. Queens and workers were anesthetized on ice and the cuticle of the head was removed. The dissected heads were transferred into Tissue-Tek OCT compound (Sakura Fine Technical, Tokyo, Japan), then frozen on dry ice and stored at –80°C until use. The procedures for frozen-brain sections and detection with the DIG-labeled riboprobes by *in situ* hybridization were performed as described previously [49]. The sense or antisense riboprobes corresponding to 1028-bp fragments within the open reading frame (ORF) of *Amddc* were synthesized by *in vitro* transcription with a DIG RNA Labeling Mix (Roche, Basel, Switzerland). The primer sequences for the riboprobe synthesis were as follows: Fw: 5’-GTGATGTTGGACTGGCTAGG-3’, Rv: 5’-TGGATGTCTTTGCTCTCGCT-3’. Sense probes were used as a negative control in each experiment. The intensity and brightness of the micrographs were processed with Photoshop (Adobe Systems, San Jose, CA, USA).

### Statistics

The amount of dopamine and NADA in the brains were compared between queens and workers among different days before eclosion by a one-way ANOVA following the post hoc test (Fisher’s PLSD, P = 0.05 significant level) for multiple comparisons. The relative expression of dopamine-related genes in the brains was also compared between castes among different days before eclosion by using the same statistical test.

## Results

### Dynamics of dopamine levels in the brain

**Fig 1:**
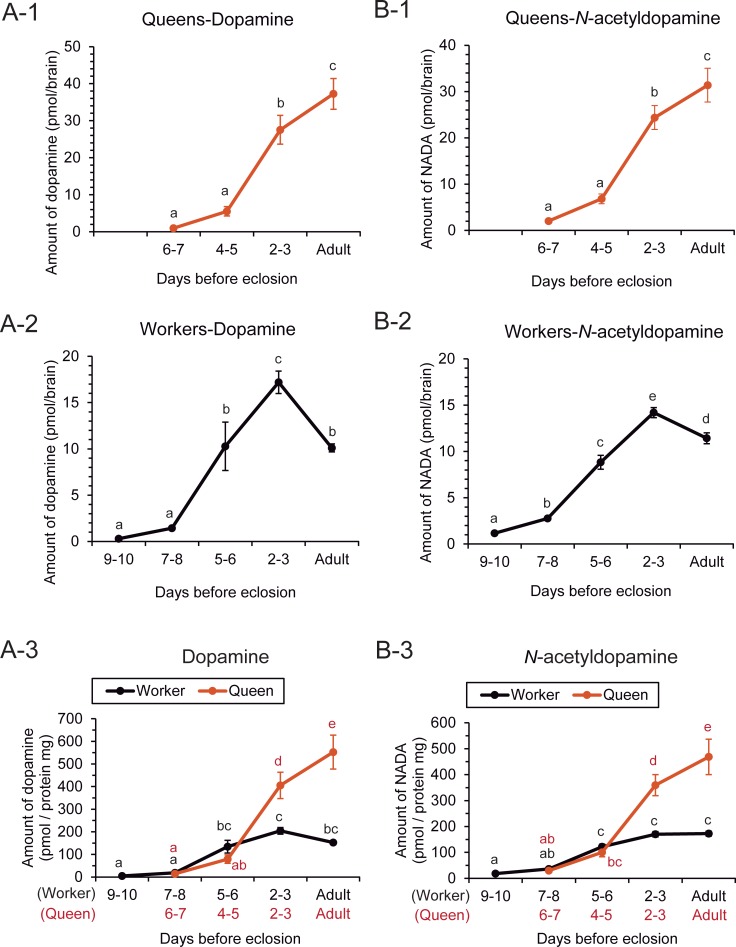
Brain levels of dopamine and *N*-acetyldopamine (NADA) in queens and workers from pupation to eclosion. The brain levels of dopamine (A) and NADA (B) per brain are indicated as days before eclosion in queens (A-1 and B-1) and workers (A-2 and B-2), respectively. The brain levels of dopamine (A-3) and NADA (B-3) per protein mg are shown as days before eclosion to compare between castes. Different letters beside the plotted points indicate significant differences between the groups (Fisher’s PLSD post hoc test, P <0.05). Each plotting point and error bar indicates mean and standard error (N = 10), respectively.

**Fig 2:**
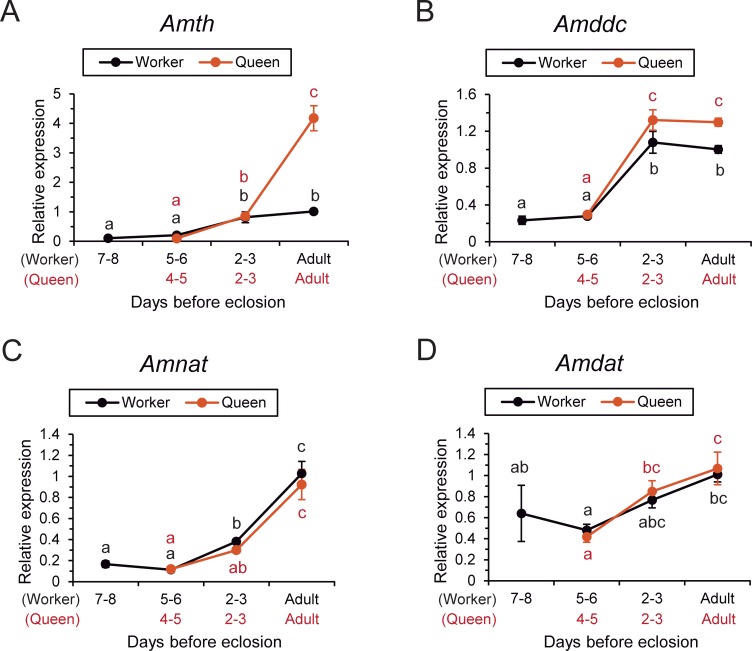
Relative expression levels of dopamine-related genes in queens and workers. The expression levels of genes encoding enzymes involved in dopamine biosynthesis [*Amth* (A) and *Amddc* (B)] and in dopamine conversion [*Amnat* (C)] and a dopamine transporter gene [*Amdat* (D)] are indicated. Different letters beside the plotted points indicate significant differences between the groups (Fisher’s PLSD post hoc test, P <0.05). Each plotting point and error bar indicates mean and standard error (N = 5), respectively.

In each caste, the dopamine levels per brain differed significantly among the days before eclosion that we examined (one-way ANOVA, queens: d.f. = 3, F = 35.39, P <0.001, N = 40, ten samples each; workers: d.f. = 4, F = 28.87, P <0.001, Fisher’s PLSD post hoc test, P <0.05, N = 50, ten samples each). In queens, dopamine levels in the brain 6–7 days before eclosion were on average 0.956 pmol/brain and were highest on the day of eclosion (37.247 pmol/brain on average) during the pupal stage (Fig 1A-1). However, in workers, dopamine levels in the brain 9–10 days before eclosion were on average 0.3 pmol/brain and highest 2–3 days before eclosion (17.197 pmol/brain on average) during the pupal stage and were significantly lower on the day of eclosion (10.1 pmol/brain on average) than 2–3 days before eclosion (Fig 1A-2).

**Fig 3:**
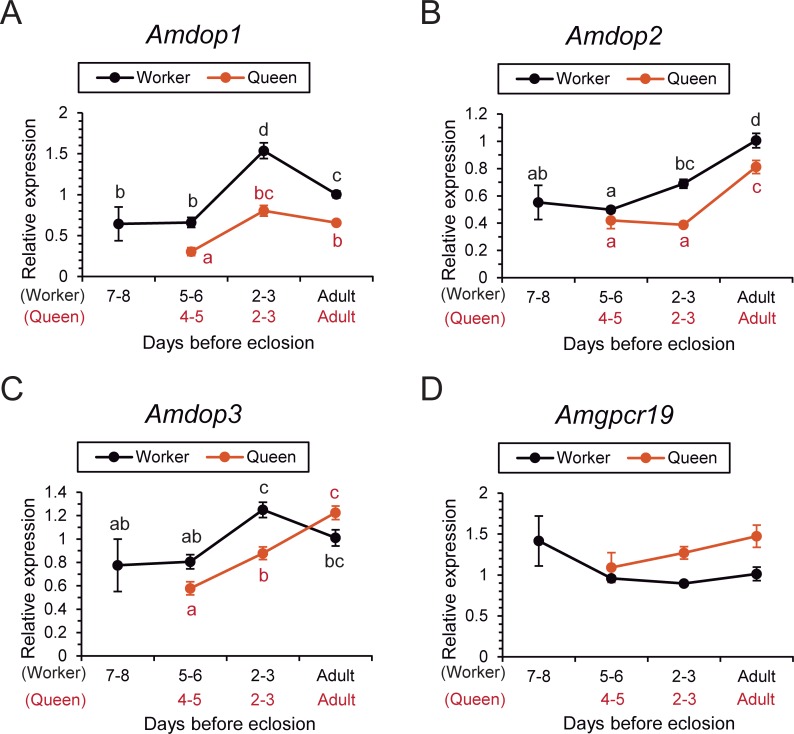
Relative expression levels of dopamine receptor genes in queens and workers. The expression levels of dopamine receptor genes [*Amdop1* (A), *Amdop2* (B), and *Amdop3* (C)] and those of a dopamine-ecdysteroid receptor gene [*Amgpcr19* (D)] are indicated. Different letters beside the plotted points indicate significant differences between the groups (Fisher’s PLSD post hoc test, P <0.05). Each plotting point and error bar indicates mean and standard error (N = 5), respectively.

To examine any caste differences in dopamine levels in the brain, the levels were normalized to the protein content of a whole brain and then compared with the day of eclosion. In both queens and workers, the dopamine levels per protein (mg) in the brain differed significantly across the pre-eclosion times examined (one-way ANOVA, d.f. = 8, F = 30.793, P <0.001, N = 90, ten samples each, Fig 1A-3). On the day of eclosion, the dopamine levels were significantly (3.6-fold) higher in queens than in workers (Fisher’s PLSD post hoc test, P <0.05). Caste differences were also detected 2–3 days before eclosion, but not 4–5 (5–6) days or 6–7 (7–8) days before eclosion (Fig 1A-3).

Trends in brain NADA levels were similar with those seen for dopamine. In each caste, the NADA levels per brain differed significantly among the days before eclosion that we examined (one-way ANOVA, queens: d.f. = 3, F = 36.78, P <0.001, N = 40, ten samples each; workers: d.f. = 4, F = 124.47, P <0.001, N = 50, ten samples each; Fisher’s PLSD post hoc test, P <0.05). In queens, the NADA levels per brain 6–7 days before eclosion were on average 2.01 pmol/brain and were highest on the day of eclosion (31.386 pmol/brain on average) during the pupal stage (Fig 1B-1). In contrast, the dopamine levels per brain in workers 9–10 days before eclosion were on average were 1.169 pmol/brain, were highest 2–3 days before eclosion (14.195 pmol/brain on average) during the pupal stage and were significantly lower on the day of eclosion (11.423 pmol/brain on average) than 2–3 days before eclosion (Fig 1B-2). Among the days before eclosion that we examined, the NADA levels per protein mg in the brains of both castes differed significantly (one-way ANOVA, d.f. = 8, F = 30.812, P <0.001, N = 90, ten samples each, Fig 1B-3). The caste differences in NADA levels were detected on the day of eclosion and 2–3 days before eclosion (Fisher’s PLSD post hoc test, P <0.05), but not at 4–5 (5–6) days and 6–7 (7–8) days before eclosion (Fig 1B-3).

### Transcription levels of dopamine-related genes in the brains

#### Genes encoding enzymes involved in the dopamine metabolic pathway, and a dopamine transporter gene

The relative expression of genes encoding enzymes involved in the biosynthesis of dopamine (*Amth* and *Amddc*), in the degradation, or conversion, of dopamine into NADA (*Amnat*), and a dopamine transporter (*Amdat*) were quantified and compared between castes across pupal development until eclosion.

For *Amth*, the relative expression in the brain differed significantly among the days examined in both queens and workers (one-way ANOVA, d.f. = 6, F = 60.403, P <0.001, N = 35, five samples each, Fig 2A). In each caste, the levels were significantly higher on the day of eclosion (newly emerged adults) and 2–3 days before eclosion than at 4–5 (5–6) days before eclosion (Fisher’s PLSD post hoc test, P <0.05). *Amth* expression levels were significantly higher in queens than in workers on the day of eclosion (Fisher’s PLSD, P <0.05), but did not differ between castes at 2–3 and 4–5 (5–6) days before eclosion (Fig 2A).

In terms of *Amddc*, the relative expression in the brain differed significantly among the days examined in both queens and workers (one-way ANOVA, d.f. = 6, F = 52.356, P <0.001, N = 35, five samples each, Fig 2B). In each caste, the levels were significantly higher on the day of eclosion and 2–3 days before eclosion than at 4–5 (5–6) days before eclosion (Fisher’s PLSD, P <0.05). *Amddc* expression was significantly higher in queens than in workers on the day of eclosion and 2–3 days before eclosion (Fisher’s PLSD, P <0.05), but not at 4–5 (5–6) days before eclosion (Fig 2B).

The relative expression of *Amnat* in the brain differed significantly as pupae developed into adults of both castes (one-way ANOVA, d.f. = 6, F = 28.181, P <0.001, N = 35, five samples each, Fig 2C). In each caste, the levels were significantly higher on the day of eclosion than at 2–3 and 4–5 (5–6) days before eclosion (Fisher’s PLSD, P <0.05). In workers, the levels at 2–3 days before eclosion were intermediate between 5–6 days before eclosion and the day of eclosion. No caste differences in *Amnat* expression were detected from pupae to eclosion (Fisher’s PLSD, P >0.05, Fig 2C).

The relative expression of *Amdat* in the brain differed significantly as pupae developed into adults of both castes (one-way ANOVA, d.f. = 6, F = 3.589, P <0.01, N = 35, five samples each, Fig 2D). In each caste, the levels were significantly higher on the day of eclosion than at 4–5 (5–6) days before eclosion (Fisher’s PLSD, P <0.05) and the levels at 2–3 days were intermediate at 4–5 (5–6) days before eclosion and the day of eclosion. No caste differences in *Amdat* expression were detected from pupae to eclosion (Fisher’s PLSD, P >0.05, Fig 2D).

Thus, the enhanced expression of genes encoding enzymes involved in dopamine synthesis was detected in queens, but not of enzymes involved in dopamine conversion or of the dopamine transporter.

#### Dopamine receptor genes

The relative expression of dopamine receptor genes (*Amdop1*, *Amdop2*, and *Amdop3*) and a dopamine/ecdysteroid receptor gene (*Amgpcr19*) were quantified and compared between castes as above.

The relative expression of *Amdop1* in the brain differed significantly across pupal development in both castes (one-way ANOVA, d.f. = 6, F = 16.231, P <0.001, N = 35, five samples each, Fig 3A). In each caste, the levels were significantly higher on the day of eclosion and 2–3 days before eclosion than at 4–5 (5–6) days before eclosion (Fisher’s PLSD post hoc test, P <0.05). In workers, the levels at 2–3 days before eclosion were the highest. *Amdop1* expression was significantly lower in queens than in workers at 2–3 and 4–5 (5–6) days before eclosion and on the day of eclosion (Fisher’s PLSD, P <0.05, Fig 3A).

In terms of *Amdop2*, the relative expression in the brain differed significantly in both castes (one-way ANOVA, d.f. = 6, F = 13.482, P <0.001, N = 35, five samples each, Fig 3B). In queens, the levels were significantly higher on the day of eclosion than on other days (Fisher’s PLSD post hoc test, P <0.05), whereas the levels in workers were significantly higher on the day of eclosion and 2–3 days before eclosion than at 5–6 days before eclosion (Fisher’s PLSD post hoc test, P <0.05). *Amdop2* expression levels were significantly lower in queens than in workers 2–3 days before eclosion and on the day of eclosion (Fisher’s PLSD, P <0.05, Fig 3B).

The relative expression of *Amdop3* in the brain differed significantly in both castes (one-way ANOVA, d.f. = 6, F = 5.790, P <0.001, N = 35, five samples each, Fig 3C). In queens, expression on the day of eclosion was the highest among the days examined, whereas 4–5 days before eclosion it was the lowest (Fisher’s PLSD post hoc test, P <0.05). In workers, expression was significantly higher 2–3 days before eclosion than at 5–6 or 7–8 days before eclosion (Fisher’s PLSD post hoc test, P <0.05), and expression on the day of eclosion was intermediate between them. *Amdop3* expression was significantly lower in queens than in workers 2–3 days before eclosion (Fisher’s PLSD, P <0.05, Fig 3C).

For *Amgpcr19*, relative expression in the brain did not differ between castes or across pupal development (one-way ANOVA, d.f. = 6, F = 2.295, P = 0.063, N = 35, five samples each, Fig 3D).

Thus, the expression of dopamine receptors was downregulated in queens from pupae to eclosion in *Amdop1* and *Amdop2*, and at 2–3 days before eclosion in *Amdop3*, but dopamine/ecdysteroid receptor *Amgpcr19* did not exhibit any between caste differences.

### Distribution of cells expressing *Amddc* in the brain

**Fig 4:**
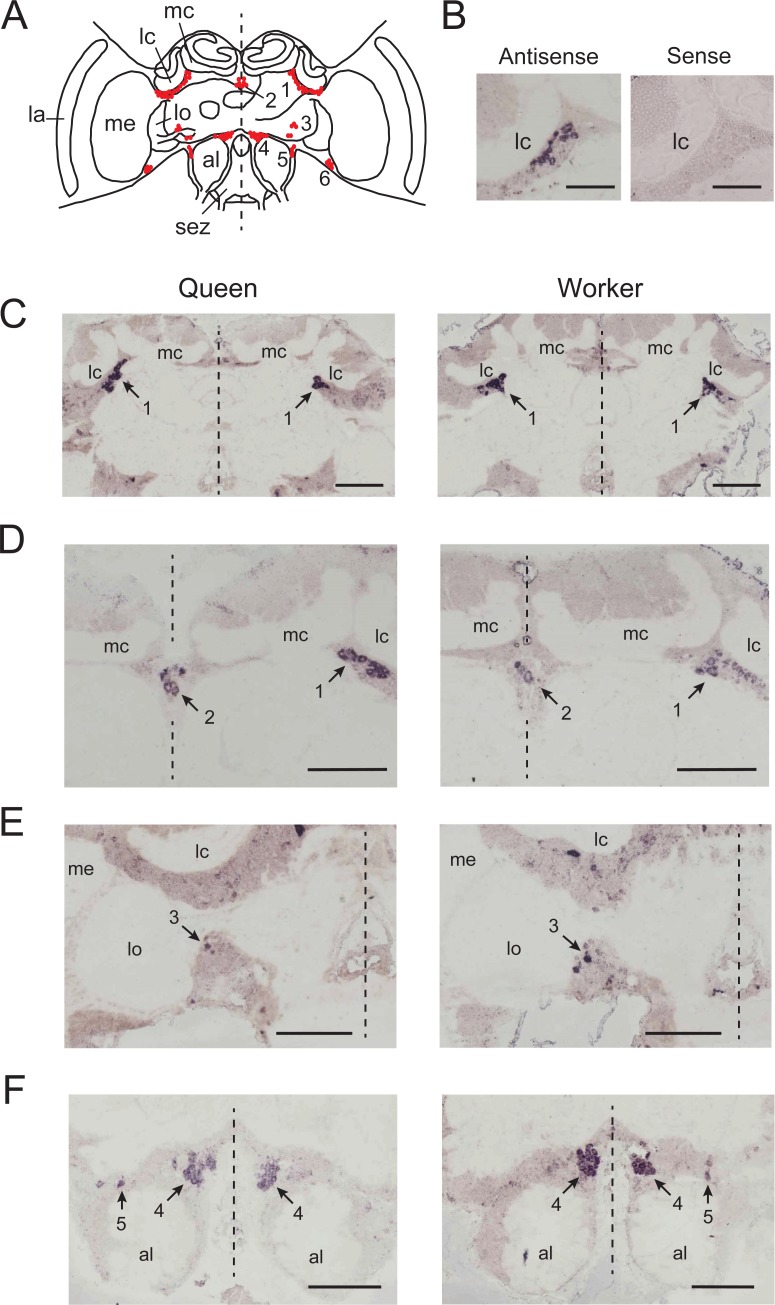
Cells expressing *Amddc* stained by *in situ* hybridization in the brain of queens and workers. (A) Soma clusters of dopaminergic cells in the brain based on previous reports (1–5) and a novel soma cluster in the posterior region of the medulla in the optic lobe found in the current study (6). (B) Somata stained with antisense *Amddc* riboprobes but not stained with sense riboprobes. Scale bars: 0.1 mm. (C–F) Soma clusters stained with antisense *Amddc* riboprobes. Dotted lines indicate the midline of the brain. Scale bars: 0.2 mm. al: antennal lobe, cb: central body, la: lamina, lc: lateral calyx of mushroom body, lo: lobula, mc: median calyx of mushroom body, me: medulla, ot: optic tubercle, sez: subesophageal zone.

**Fig 5:**
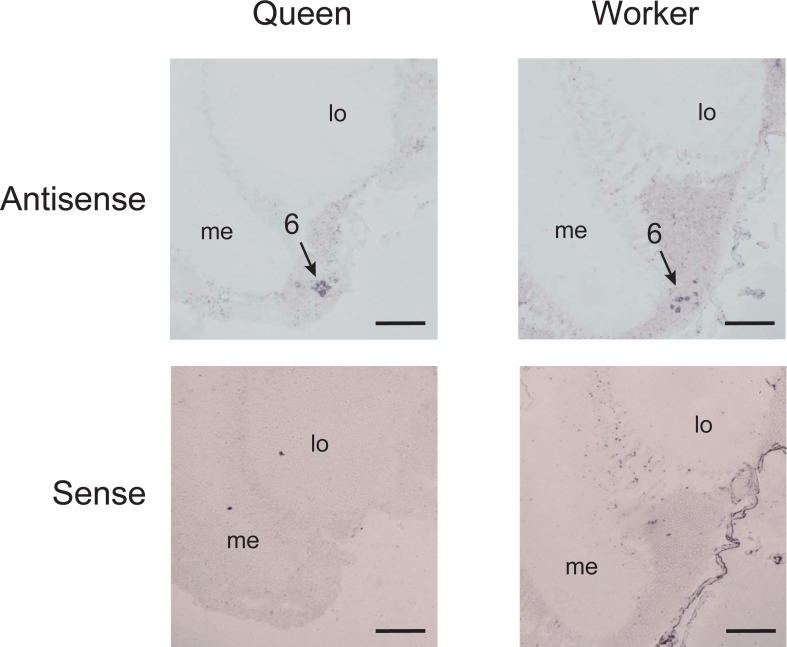
Cells expressing *Amddc* in the posterior region of the medulla in the optic lobe. The soma clusters stained with antisense *Amddc* riboprobes were observed in the brain of both queens and workers, whereas no somata were stained with sense *Amddc* reboprobes. The position of the soma cluster corresponds to cluster 6 in Fig 4A. Scale bars: 0.1 mm.

*In situ* hybridization using antisense *Amddc* riboprobes detected somata of cells in particular brain regions (four brains each from queens and workers, Figs 4 and 5), most of which corresponded with the location of soma clusters previously described in worker dopaminergic cells [50–52]. These clusters were thus numbered 1 to 5 based on previous reports [50–52] (Fig 4A). The cytoplasm of somata were selectively stained with antisense *Amddc* reboprobes, whereas staining of somata nuclei was either weak or undetectable (Fig 4B). Somata treated with sense *Amddc* reboprobes were not stained (Fig 4B and S1 Fig), indicating that the staining of cells expressing *Amddc* was not an artifact. Some somata detected by the antisense riboprobes were located in the ventral region of the medulla on both sides of the optic lobes (Figs 4A and 5), which have not previously been reported as dopamine-like immunoreactive cells [50,51] or tyrosine hydroxylase-immunoreactive cells [52] in honey bee workers. This pair of somata in the optic lobes were stained in the brains of both queens and workers (four brains each; Fig 5). In the subesophageal zone, there were several *Amddc*-expressing cells that corresponded to previously reported somata positions [50,52] (S2 Fig). These cells were stained with antisense riboprobes across sections in both queens and workers, but could not be identified on the soma positions.

The locations of somata expressing *Amddc* in the brains of same-aged queens (newly emerged adults) and workers (7-day-old pupae) were similar (Figs 4C–4F and 5). The precise number of somata expressing *Amddc* in each cluster could not be determined because the sequential frozen sections containing different somata were allocated differently for the antisense and sense probes. However, the number of somata stained with the antisense riboprobes were approximately similar between queens and workers. There were no unstained soma clusters in either caste.

## Discussion

The current study described the dynamics of dopamine levels, the relative expression of genes encoding enzymes involved in dopamine synthesis, dopamine conversion, a dopamine transporter, and dopamine receptors in the brain during metamorphosis in both queens and workers of the honey bee. Our results showed that differences between castes could be detected in both dopamine synthesis and receptor expression, but that the trends differed: queens showed high expression of genes encoding enzymes involved in dopamine synthesis but lower expression of genes encoding certain dopamine receptors. These results indicate that different neuroendocrine states in the brains of different castes occur during metamorphosis.

The levels of dopamine and its metabolite (NADA) in the brain increased significantly in early stage pupae (i.e. 2–5 days old) of both queens and workers. Dopamine levels in queens increased until eclosion, whereas those in workers increased until 5–6 days before eclosion. On the day of eclosion, dopamine levels in queens were 3.6-fold higher than those in workers. These caste-specific dynamics in dopamine levels can be explained by the expression of *Amddc* and *Amth*. The former was more highly expressed in queens from 2–3 days before eclosion, whereas the latter was expressed more highly in queens on the day of eclosion. The differential timing of expression might account for the continuous increase in brain dopamine levels without a peak during metamorphosis in queens: the increase in *Amddc* expression might initially increase dopamine levels and decrease DOPA levels, and the increased *Amth* expression might subsequently supply DOPA for dopamine synthesis. In contrast, the expression of *Amnat* was similar between castes and increased until eclosion. This indicated that caste differences in dopamine levels are strongly associated with the expression of *Amth* and *Amddc*, but not of *Amnat*. Furthermore, the release and conversion of dopamine might occur in both queens and workers in parallel with an increase in dopamine, the higher dopamine release in queens in particular would result in the higher dopamine conversion of queens and in the caste differences of the brain NADA levels.

The dopamine transporter is a membrane protein involved in the uptake of dopamine from presynaptic dopaminergic cells or other cells. Given that the levels of dopamine and NADA were increased in queen brains, active dopamine release and conversion in queens would be expected. Therefore, we also expected that the dopamine uptake system would be active, and that the dopamine transporter gene *Amdat* would be expressed more in queens than in workers. However, *Amdat* expression did not differ between castes and became elevated in response to eclosion in both queens and workers. The functional role(s) of *Amdat* during the pupal stage remain unknown.

The distribution of cells expressing *Amddc* in the brain revealed that the positions of somata in the dopaminergic cells of queens and workers were similar during stages when the levels of dopamine were the highest. Although dopamine levels in the brain were higher in queens than in workers, there were no additional clusters of cells expressing *Amddc* in queen brains. Therefore, some of these dopaminergic cells might differentially express *Amddc* in the brains of queens. It is also possible that the increased dopaminergic cell number in queens accounts for the higher dopamine levels. However, the number of dopaminergic cells was not determined in this study and the possibility remains to be tested. Several somata in dopaminergic cells located in the posterior medulla of a pair of optic lobes were identified as novel dopaminergic cells. These cells were detected in the brains of both queens and workers. However, we do not know whether the cells are neurons or glial cells, or what functional role the cells have in the brains. More detailed investigations of the morphology of the cells is required.

The expression of dopamine receptor genes differed between castes. Queens showed reduced expression of genes encoding dopamine receptors compared with workers from 5–6 days before eclosion to newly emerged adults in terms of *Amdop1*, at 2–3 days before eclosion and emerged adults in terms of *Amdop2*, and at 2–3 days before eclosion in terms of *Amdop3*. It was interesting that both D1-like (*Amdop1* and *Amdop2*) and D2-like (*Amdop3*) receptors were depressed in queens compared with workers, although the actions of these types of receptor are excitatory and inhibitory at the cellular level, respectively [53]. These differences in gene expression contrast with the trends in dopamine levels and expression of enzymes involved in dopamine synthesis, and thus might account for the elevated dopamine in queens, likely as a negative feedback mechanism. In mammals, both D1-like and D2-like receptors reduce dopamine biosynthesis and release [54–56]. D1-like receptors can act indirectly on dopaminergic neurons negatively, whereas D2-like receptors can directly regulate dopaminergic neurons mediated by autoreceptors. Genetic knockout of D2-like autoreceptors increases dopamine synthesis and release [55]. Although there are few studies on autoreceptors in insect brains, the high dopamine levels in honey bee queens might be associated with the reduced expression of both D1-like and D-2-like receptors. It is also possible that the caste differences in the receptor expression levels might result from different proportions of the subcompartments in the brain between castes [12,13]. For example, target tissues expressing dopamine receptors in brain subcompartments might be proportionally smaller in queens than in workers, resulting in a relative reduction in receptor gene expression in queens. To investigate this further, histological investigations of dopamine receptors between castes are required. In terms of *Amgpcr19*, which encodes a dopamine-ecdysteroid membrane receptor, its expression did not differ significantly between castes. Although the hemolymph ecdysteroid titer in both queens and workers changes significantly during metamorphosis [57], the expression of *Amgpcr19* was stable during this stage, suggesting that activation of this receptor by ecdysteroid or dopamine does not differ between castes.

The roles of dopamine during the pupal stage in the honey bee are largely unknown. There are several reports implicating dopamine after eclosion in virgin queens, which have high levels of dopamine in the brain [18,31]. Application of a dopamine antagonist can result in decreased aggression against rival virgin queens under appropriate concentrations of the drug [32]. Dopamine receptor drugs can also control locomotor activities in virgin queens [31]. These results suggest that dopamine has a role in enhancing behavioral activities of virgin queens, including aggression against rival virgin queens and mating flight activities. In particular, the aggression against rival queens requires high levels of dopamine immediately after eclosion. As for the endocrine state at eclosion, the higher dopamine levels in queens compared to workers might result from the increased expression of *Amth* and *Amddc* during the late pupal stages. In *Drosophila melanogaster*, experimental transient activation of dopaminergic neurons in brains of late stage pupae increased locomotor activity and visual responsiveness in adults [58]. Thus, activation of dopaminergic neurons in the brain during metamorphosis significantly influenced adult behavior, including that of honey bee queens.

The promotion of reproduction including ovarian activity and oviposition in adult females is an important role of dopamine in solitary [25–27] and eusocial insects [27–30]. In honey bees, the expression of dopamine receptor genes in adult ovarian tissues has been reported [59]. The enhanced dopamine levels in queen pupae might contribute to the reproduction in adult queens as well as aggressive behaviors. Dopamine might also affect the development of the reproductive system in pupae, but little is known about the effects of dopamine during the pupal stage. Since there are positive relationships between brain and hemolymph dopamine levels in honey bee queens [18], hemolymph dopamine in queen pupae may increase with brain dopamine levels and potentially act on the reproductive system. To elucidate this possibility, studies on the effects of dopamine on reproductive tissues and dopamine receptor expression during the pupal stage are required.

The present study revealed caste differences in the dopaminergic system during metamorphosis in the honey bee. The caste-specific dopamine dynamics in the brain might derive from the differential expression of genes encoding enzymes involved in dopamine biosynthesis. The distribution of cells expressing *Amddc* in the brain were similar between castes, suggesting that the expression of *Amddc* is upregulated in some cells in queens but not by the addition of cell clusters. In contrast, dopamine receptor genes were downregulated in queens or showed similar expression levels between castes, suggesting the influence of excessive dopamine production in the brain of queens. These caste-specific ligand–receptor characters might generate the caste-specific behavior and intensive division of reproduction seen in these highly eusocial honey bees.

## Supporting information

S1 Fig Sections of brain tissues stained by *in situ* hybridization using sense *Amddc* riboprobes. Sequential frozen sections were arranged in parallel for antisense and sense *Amddc* riboprobes. Dotted lines indicate the midline of the brain. Each section corresponds to photos in Fig 4. Scale bars: 0.2 mm.
(EPS)

S2 Fig Cells expressing *Amddc* stained by *in situ* hybridization in the subesophageal zone. The soma clusters stained with antisense *Amddc* riboprobes were observed in the zone in both queen and worker (arrows), whereas no somata were stained with sense *Amddc* riboprobes. Dotted lines indicate the midline of the brain. Scale bars: 0.1 mm.
(EPS)

S1 Table Primer sequences for target and reference genes. (PDF)

S2 Table Comparison of variation between biological replicates and technical replicates. (PDF)

S3 Table Correlation coefficients (R^2^) and slopes of standard curves, and amplification efficiencies of qPCR for each gene. (PDF)

S4 Table Levels of dopamine and *N*-acetyldopamine in brains. (PDF)

S5 Table Cq-values for each gene and relative expression level calculated by the ΔΔCq method. (PDF)
