# Supplementary material for: Caste-specific development of the dopaminergic system during metamorphosis in female honey bees
Source: PLoS One. 2018 Oct 29;13(10):e0206624. doi: 10.1371/journal.pone.0206624 (PMC6205643; doi:10.1371/journal.pone.0206624)
Supplement: S1 Table — (PDF) [file pone.0206624.s003.pdf]

S1 Table. Primer sequences in the target genes and reference genes.

Gene	Primer sequence		Remarks
Target			
Amth	Forward	TACTTCGTCGCGGATAGCTT	Tyrosine hydroxylase
	Reverse	CCACGCTGTCCAGTATCTCA	
Amddc	Forward	ATTGCTTGCATGGGATTCTC	DOPA decarboxylase
	Reverse	GCAGTTCCCTGAATGACACC	
Amnat	Forward	GATCCTCCGAATGACGAAGA	Dopamine *N*-acetyltransferase
	Reverse	CGCTTTGGCAACTCCTTTAC	
Amdat	Forward	TGGCTACTCGATGCTGTTTG	Dopamine transporter
	Reverse	TATCGGAGCAACGAATTTCC	
Amdop1	Forward	TGAACGATCTCCTCGGCTAT	Dopamine receptor 1 (D1-like), Ref. 1
	Reverse	ACCCAACGACCGTATCTGAG	
Amdop2	Forward	CTGCCCCTGTTCGTAATGGT	Dopamine receptor 2 (D1-like)
	Reverse	GCTTGGTGCCCAATTTCAGG	
Amdop3	Forward	CGGTTCAGGATCCCGTTTCA	Dopamine receptor 3 (D2-like)
	Reverse	TCCTTCTTCGCCGAGCTTTT	
Amgpcr19	Forward	GGATATCGTGTGTCGTCTCG	Dopamine-ecdysteroid receptor, Ref. 1
	Reverse	CTCGTAACGCAATGGTTTCC	
Reference			
Amact	Forward	TGCCAACACTGTCCTTTCTG	Actin, Ref. 2
	Reverse	AGAATTGACCCACCAATCCA	
Amrp49	Forward	CGTCATATGTTGCCAACTGGT	Ribosomal protein 49, Ref. 2
	Reverse	TTGAGCACGTTCAACAATGG	
Amef1a	Forward	GGAGATGCTGCCATCGTTAT	Elongation factor 1-alpha, Ref. 2
	Reverse	CAGCAGCGTCCTTGAAAGTT	
Amtbpaf	Forward	TTGGTTTCATTAGCTGCACAA	Tbp-association factor, Ref. 2
	Reverse	ACTGCGGGAGTCAAATCTTC	

Ref. 1: Geddes *et al*. 2013, Ref. 2: Lourenço *et al*., 2008
