# Supplementary material for: Caste-specific development of the dopaminergic system during metamorphosis in female honey bees
Source: PLoS One. 2018 Oct 29;13(10):e0206624. doi: 10.1371/journal.pone.0206624 (PMC6205643; doi:10.1371/journal.pone.0206624)
Supplement: S2 Table — (PDF) [file pone.0206624.s004.pdf]

S2 Table. Comparison of variations between biological replicates and technical replicates.

Amtbpaf	Queens			Workers				Paired t-test
	2-3 days	4-5 days	7 days (adult)	2-3 days	4-5 days	7-8 days	10 days (adults)	mean
Cq-mean	16.780858	17.110123	17.236633	17.032024	16.685341	16.874832	17.131049	
Sample variance of average Cqs	0.0375475	0.0109391	0.0107731	0.4696633	0.0359074	0.0225568	0.0956731	0.09758
Mean square of differences between duplicate Cqs	0.0217166	0.0049908	0.0610841	0.042748	0.0106456	0.0514601	0.0011605	0.0276865
								t = 1.128
								P = 0.151

Amth	Queens			Workers				Paired t-test
	2-3 days	4-5 days	7 days (adult)	2-3 days	4-5 days	7-8 days	10 days (adults)	mean
Cq-mean	23.455344	20.677235	18.471958	23.889043	22.281726	20.588908	20.397154	
Sample variance of average Cqs	0.1183071	0.2038665	0.1186782	0.2633902	0.0255409	0.4195804	0.0858128	0.1764537
Mean square of differences between duplicate Cqs	0.315476	0.1351617	0.2997207	0.4408318	0.0676087	0.0031852	0.0456913	0.1868108
								t = -0.126
								P = 0.452

Amddc	Queens			Workers				Paired t-test
	2-3 days	4-5 days	7 days (adult)	2-3 days	4-5 days	7-8 days	10 days (adults)	mean
Cq-mean	20.369752	18.524248	18.659611	21.006649	20.327243	18.604733	18.927796	
Sample variance of average Cqs	0.0696865	0.1103055	0.0283844	0.0682377	0.0506896	0.075076	0.0654962	0.0668394
Mean square of differences between duplicate Cqs	0.0924852	0.0057672	0.0100781	0.0762951	0.0097705	0.0006468	0.0102551	0.0293283
								t = 2.195
								P < 0.05

Amnat	Queens			Workers				Paired t-test
	2-3 days	4-5 days	7 days (adult)	2-3 days	4-5 days	7-8 days	10 days (adults)	mean
Cq-mean	17.99582	16.965802	15.508571	17.794021	17.936466	16.378232	15.227525	
Sample variance of average Cqs	0.2161067	0.0880328	0.1975236	0.1113047	0.0284804	0.0080605	0.2134895	0.1232855
Mean square of differences between duplicate Cqs	0.0009913	0.0063505	0.7004607	0.0054555	0.0034492	0.0012359	0.9199951	0.2339912
								t = -0.839
								P = 0.217

Amdat	Queens			Workers				Paired t-test
	2-3 days	4-5 days	7 days (adult)	2-3 days	4-5 days	7-8 days	10 days (adults)	mean
Cq-mean	23.131237	22.44618	22.252999	23.088716	22.846015	22.336935	22.186944	
Sample variance of average Cqs	0.1087462	0.1977222	0.2259516	0.0920965	0.0977997	0.0607112	0.0157048	0.1141046
Mean square of differences between duplicate Cqs	0.0052057	0.0121589	0.0951588	0.0710725	0.0145585	0.0395882	0.0008096	0.0340789
								t = 3.250
								P < 0.01

Amdop1	Queens			Workers				Paired t-test
	2-3 days	4-5 days	7 days (adult)	2-3 days	4-5 days	7-8 days	10 days (adults)	mean
Cq-mean	20.967635	19.84544	20.244577	20.291315	19.708772	18.667943	19.531962	
Sample variance of average Cqs	0.1952891	0.0746921	0.0163908	0.0581213	0.0674471	0.0322993	0.1422133	0.083779
Mean square of differences between duplicate Cqs	0.3202904	0.0074356	0.0111762	0.0514723	0.0204974	0.0042198	0.006159	0.0601787
								t = 0.786
								P = 0.231

Amdop2	Queens			Workers				Paired t-test
	2-3 days	4-5 days	7 days (adult)	2-3 days	4-5 days	7-8 days	10 days (adults)	mean
Cq-mean	22.001704	22.39734	21.461148	21.923713	21.610445	21.334122	21.045693	
Sample variance of average Cqs	0.153403	0.0156574	0.0134714	0.0586112	0.0723889	0.011068	0.193253	0.073979
Mean square of differences between duplicate Cqs	0.0404636	0.0045869	0.143193	0.0211213	0.0614087	0.00635	0.0032797	0.0400576
								t = 0.903
								P = 0.201

Amdop3	Queens			Workers				Paired t-test
	2-3 days	4-5 days	7 days (adult)	2-3 days	4-5 days	7-8 days	10 days (adults)	mean
Cq-mean	22.662794	22.375962	22.017806	22.657862	22.080537	21.628663	22.19812	
Sample variance of average Cqs	0.0785666	0.0708738	0.025157	0.0692292	0.0428097	0.0131525	0.1336783	0.0619239
Mean square of differences between duplicate Cqs	0.0447814	0.0534218	0.1085339	0.0037974	0.0010794	0.0029925	0.0138489	0.0326365
								t = 1.255
								P = 0.128

Amgpcr19	Queens			Workers				Paired t-test
	2-3 days	4-5 days	7 days (adult)	2-3 days	4-5 days	7-8 days	10 days (adults)	mean
Cq-mean	18.858181	18.900376	18.82472	18.759012	18.871974	19.158063	19.254824	
Sample variance of average Cqs	0.2206021	0.0246905	0.0426818	0.0567586	0.0415513	0.0361598	0.0550216	0.0682094
Mean square of differences between duplicate Cqs	0.0237938	0.0013521	0.0147588	0.0555309	0.0090738	0.0133116	0.0029494	0.0172529
								t = 2.041
								P < 0.05

An average Cq from duplicate Cqs in an individual sample was used for calculation of “Sample variance of average Cqs”. “Sample variance of average Cqs” was calculated as $\{(Cq_{1\ average} - Cq_{mean})^2 + (Cq_{2\ average} - Cq_{mean})^2 + (Cq_{3\ average} - Cq_{mean})^2 + (Cq_{4\ average} - Cq_{mean})^2 + (Cq_{5\ average} - Cq_{mean})^2\} / 5$, indicating variation of biological

replicates. “Mean square of differences between duplicate Cqs” was calculated as $\{(Cq_{1\ large} - Cq_{1\ small})^2+(Cq_{2\ large} – Cq_{2\ small})^2+(Cq_{3\ large} – Cq_{3\ small})^2+(Cq_{4\ large} – Cq_{4\ small})^2+(Cq_{5\ large} – Cq_{5\ small})^2\} / 5$, indicating variation of technical replicates.
