# Supplementary material for: Caste-specific development of the dopaminergic system during metamorphosis in female honey bees
Source: PLoS One. 2018 Oct 29;13(10):e0206624. doi: 10.1371/journal.pone.0206624 (PMC6205643; doi:10.1371/journal.pone.0206624)
Supplement: S3 Table — (PDF) [file pone.0206624.s005.pdf]

S3 Table. Correlation coefficients (R^2) and slopes of standard curves, and amplification efficiencies of qPCR in each gene.

	Genes								
	Amtbpaf	*Amth*	*Amddc*	*Amnat*	*Amdat*	*Amdop1*	*Amdop2*	*Amdop3*	*Amgpcr*
Correlation coefficient (R^2)	0.997	0.99	0.997	0.997	0.996	0.983	0.992	0.996	0.996
Slope of standard curve	-3.204	-3.05	-3.072	-3.156	-3.279	-3.203	-3.169	-3.113	-3.116
Amplification efficiency	1.052	1.128	1.116	1.074	1.018	1.052	1.068	1.095	1.094
