# Supplementary material for: Caste-specific development of the dopaminergic system during metamorphosis in female honey bees
Source: PLoS One. 2018 Oct 29;13(10):e0206624. doi: 10.1371/journal.pone.0206624 (PMC6205643; doi:10.1371/journal.pone.0206624)
Supplement: S4 Table — (PDF) [file pone.0206624.s006.pdf]

S4 Table. Data of brain levels of dopamine and *N*-acetyldopamine.

	Dopamine (pmol / brain)									Dopamine (pmol / protein mg)									
	Queens				Workers					Queens				Workers					
	0-1 day	2-3 days	4-5 days	7 days (adult)	0-1 day	2-3 days	4-5 days	7-8 days	10 days (adult)	0-1 day	2-3 days	4-5 days	7 days (adult)	0-1 day	2-3 days	4-5 days	7-8 days	10 days (adult)
	0.50700	10.68122	12.86822	40.38623	0.43028	1.49346	6.84593	13.05320	8.00956	6.70131	162.30487	204.74291	710.98366	6.20143	23.64322	103.14776	173.09097	130.41692
	0.76672	1.98669	10.08170	43.83422	0.31343	1.41950	12.71876	13.69185	11.74624	13.18265	25.95969	126.70287	761.28250	4.63213	20.13337	166.56488	165.14495	176.47732
	0.57292	2.48209	45.93352	52.76641	0.24349	1.65665	4.67265	16.45170	10.65370	9.75827	34.69043	662.18257	736.14823	3.44428	19.61591	61.92245	173.99234	176.04900
	0.52873	5.05660	39.00989	43.53776	0.25915	1.49638	31.89471	24.53552	10.82175	6.46869	85.02599	620.67613	555.17532	3.84428	27.80930	334.41475	257.25385	164.10505
	0.78111	2.10335	34.44141	39.09646	0.27713	2.22389	8.12751	15.84604	10.77751	13.36676	30.84696	533.04074	470.32253	5.08465	20.49288	92.33607	168.43024	138.34050
	0.60071	2.05150	26.36102	55.44895	0.36589	1.25036	7.88320	12.57414	7.63890	10.68166	36.23960	417.38316	977.82685	6.11802	12.02014	72.92927	141.60987	100.89391
	0.66632	4.55743	21.20459	32.60413	0.32343	1.50457	3.51543	20.38260	9.07430	12.39350	72.25188	282.32523	411.26007	4.09596	17.50767	69.14422	234.42313	134.39012
	0.73676	12.55694	27.95780	14.59631	0.32229	0.87457	14.05174	17.50688	10.73373	11.11063	193.37267	378.51443	244.70288	7.63577	13.96929	240.16652	286.45129	191.21950
	3.70602	9.64536	40.77178	19.67898	0.19286	1.01772	6.06458	16.97146	10.42573	47.60119	111.58233	558.60345	278.30975	3.29974	15.71404	98.30599	223.15845	155.03251
	0.69785	4.09176	16.76412	30.51948	0.25913	1.48749	6.95585	20.96145	11.11653	8.02504	41.88979	269.43292	379.46979	3.52608	15.52729	102.60565	219.23414	158.04421
mean	0.95641	5.52129	27.53940	37.24689	0.29871	1.44246	10.27304	17.19748	10.09979	13.92897	79.41642	405.36044	552.54816	4.78823	18.64331	134.15376	204.27892	152.49690

	N-acetyldopamine (pmol / brain)									*N*-acetyldopamine (pmol / protein mg)									
	Queens				Workers					Queens				Workers					
	0-1 day	2-3 days	4-5 days	7 days (adult)	0-1 day	2-3 days	4-5 days	7-8 days	10 days (adult)	0-1 day	2-3 days	4-5 days	7 days (adult)	0-1 day	2-3 days	4-5 days	7-8 days	10 days (adult)
	1.83759	11.94339	16.44613	36.94912	1.69113	2.75609	9.28084	12.95775	9.06713	24.28850	181.48392	261.67005	650.47461	24.37342	43.63204	139.83467	171.82534	147.63700
	2.47631	3.94313	13.13266	40.97828	1.39232	1.79424	10.53928	13.52627	15.90566	42.57639	51.52407	165.04611	711.68242	20.57694	25.44861	138.02242	163.14771	238.96912
	1.01055	4.78108	35.33687	31.65876	0.87065	3.63921	7.54786	13.45808	11.04626	17.21225	66.82185	509.42010	441.67382	12.31577	43.09077	100.02500	142.33199	182.53594
	1.86717	7.62130	25.48268	22.14776	1.08908	2.88385	13.77160	17.02940	10.69647	22.84381	128.15096	405.44829	282.41900	16.15567	53.59449	144.39465	178.55256	162.20529
	2.45523	3.85601	36.65235	38.73591	1.13100	3.73086	7.24378	11.70809	10.90301	42.01540	56.55090	567.25894	465.98517	20.75109	34.37945	82.29609	124.44733	139.95148
	1.60641	3.61049	29.39232	49.18743	1.08547	2.71729	7.39425	12.63727	9.60517	28.56481	63.77906	465.37869	867.40670	18.15005	26.12211	68.40584	142.32082	126.86425
	1.56026	9.11322	20.39473	23.72183	1.32731	2.26369	5.65120	13.80532	11.16280	29.02090	144.47765	271.54260	299.22108	16.80928	26.34097	111.15211	158.77692	165.32080
	1.30945	12.11865	26.75903	14.01457	1.05599	2.46492	11.00069	15.05467	12.03164	19.74714	186.62324	362.28457	234.95019	25.01915	39.37151	188.01928	246.32767	214.34142
	3.84693	7.18106	25.69931	17.20559	0.74471	2.18854	8.44035	15.33415	12.10340	49.41099	83.07411	352.09951	243.32990	12.74184	33.79219	136.81694	201.62945	179.97982
	2.12751	4.02898	14.53646	39.25910	1.30654	3.27467	7.39483	16.43896	11.70425	24.46568	41.24700	233.62984	488.13560	17.77862	34.18287	109.08105	171.93370	166.39992
mean	2.00974	6.81973	24.38325	31.38584	1.16942	2.77134	8.82647	14.19500	11.42258	30.01459	100.37327	359.37787	468.52785	18.46718	35.99550	121.80481	170.12935	172.42051
