# Supplementary material for: Caste-specific development of the dopaminergic system during metamorphosis in female honey bees
Source: PLoS One. 2018 Oct 29;13(10):e0206624. doi: 10.1371/journal.pone.0206624 (PMC6205643; doi:10.1371/journal.pone.0206624)
Supplement: S5 Table — (PDF) [file pone.0206624.s007.pdf]

S5 Table. Cq-values in each gene and relative expression levels calculated by a ΔΔCq method.

- *Amtbpaf* (reference gene)

Average Cq-values in *Amtbpaf*

Amtbpaf	Queens			Workers			
	2-3 days	4-5 days	7 days (adult)	2-3 days	4-5 days	7-8 days	10 days (adults)
	16.957761	17.083186	17.214711	16.946965	16.529117	16.595668	16.725241
	16.786644	17.146400	17.252536	16.721083	16.744041	16.903927	16.784798
	16.800532	17.086245	17.195977	18.372855	17.008937	16.957076	17.406052
	16.940011	17.278602	17.418700	16.569968	16.468825	16.877633	17.416366
	16.419343	16.956180	17.101243	16.549248	16.675783	17.039858	17.322785
mean	16.780858	17.110123	17.236633	17.032024	16.685341	16.874832	17.131049
SE	0.096886	0.052295	0.051897	0.342660	0.094746	0.075095	0.154655

- *Amth*

Average Cq-values in *Amth*

Amth	Queens			Workers			
	2-3 days	4-5 days	7 days (adult)	2-3 days	4-5 days	7-8 days	10 days (adults)
	23.591197	19.953062	18.225310	23.955127	22.426976	21.141065	20.040453
	23.169153	20.566082	18.337404	23.756980	22.064027	20.195564	20.218634
	22.943037	20.848987	18.077095	23.697067	22.290401	20.487337	20.697506
	23.854363	21.350260	19.029396	23.235391	22.144638	19.658524	20.238757
	23.718972	20.667783	18.690584	24.800650	22.482589	21.462048	20.790420
mean	23.455344	20.677235	18.471958	23.889043	22.281726	20.588908	20.397154
SE	0.171979	0.225758	0.172249	0.256608	0.079908	0.323875	0.146469

ΔCq = target Cq - *Amtbpaf* Cq

Amth	Queens			Workers			
	2-3 days	4-5 days	7 days (adult)	2-3 days	4-5 days	7-8 days	10 days (adults)
	6.633436	2.869876	1.010599	7.008162	5.897859	4.545397	3.315212
	6.382509	3.419682	1.084868	7.035897	5.319986	3.291637	3.433836
	6.142505	3.762741	0.881118	5.324211	5.281464	3.530262	3.291454
	6.914353	4.071658	1.610695	6.665424	5.675813	2.780891	2.822390
	7.299629	3.711604	1.589341	8.251402	5.806806	4.422191	3.467635
mean	6.674486	3.567112	1.235324	6.857019	5.596386	3.714076	3.266106

ΔΔCq = target ΔCq - average ΔCq of adult workers

Amth	Queens			Workers			
	2-3 days	4-5 days	7 days (adult)	2-3 days	4-5 days	7-8 days	10 days (adults)
	3.367331	-0.396230	-2.255507	3.742057	2.631753	1.279291	0.049107
	3.116403	0.153576	-2.181237	3.769791	2.053880	0.025532	0.167731
	2.876400	0.496636	-2.384988	2.058106	2.015358	0.264156	0.025348
	3.648247	0.805553	-1.655410	3.399318	2.409708	-0.485214	-0.443715
	4.033523	0.445498	-1.676765	4.985296	2.540701	1.156085	0.201530
mean	3.408381	0.301007	-2.030781	3.590914	2.330280	0.447970	0.000000

Relative expression = $2^{-\Delta\Delta Cq}$

Amth	Queens			Workers			
	2-3 days	4-5 days	7 days (adult)	2-3 days	4-5 days	7-8 days	10 days (adults)
	0.096902	1.316064	4.775021	0.074736	0.161348	0.411998	0.966535
	0.115311	0.899019	4.535424	0.073313	0.240836	0.982458	0.890242
	0.136181	0.708758	5.223396	0.240131	0.247353	0.832686	0.982583
	0.079757	0.572143	3.150127	0.094777	0.188194	1.399794	1.360103
	0.061064	0.734331	3.197102	0.031570	0.171859	0.448729	0.869628
mean	0.097843	0.846063	4.176214	0.102905	0.201918	0.815133	1.013818
SE	0.013145	0.128467	0.424011	0.035815	0.017772	0.182607	0.089215

- *Amddc*

Average Cq-values in *Amddc*

Amddc	Queens			Workers			
	2-3 days	4-5 days	7 days (adult)	2-3 days	4-5 days	7-8 days	10 days (adults)
	20.478383	18.217619	18.602062	20.948186	20.009295	19.059174	18.564274
	20.019536	18.677160	18.539426	21.087774	20.520509	18.367291	18.725598
	20.117770	18.647018	18.580988	21.468666	20.639358	18.504166	19.292049
	20.736288	18.997200	18.994096	20.776008	20.230037	18.327894	19.055682
	20.496782	18.082244	18.581484	20.752612	20.237017	18.765141	19.001377
mean	20.369752	18.524248	18.659611	21.006649	20.327243	18.604733	18.927796
SE	0.131991	0.166061	0.084238	0.130612	0.112572	0.137000	0.127961

ΔCq = target Cq - *Amtbpaf* Cq

Amddc	Queens			Workers			
	2-3 days	4-5 days	7 days (adult)	2-3 days	4-5 days	7-8 days	10 days (adults)
	3.520622	1.134433	1.387351	4.001221	3.480178	2.463506	1.839033
	3.232892	1.530760	1.286891	4.366690	3.776468	1.463364	1.940800
	3.317238	1.560773	1.385011	3.095810	3.630421	1.547090	1.885996
	3.796277	1.718598	1.575396	4.206041	3.761212	1.450261	1.639315
	4.077439	1.126065	1.480241	4.203364	3.561235	1.725284	1.678592
mean	3.588894	1.414126	1.422978	3.974625	3.641903	1.729901	1.796747

ΔΔCq = target ΔCq - average ΔCq of adult workers

Amddc	Queens			Workers			
	2-3 days	4-5 days	7 days (adult)	2-3 days	4-5 days	7-8 days	10 days (adults)
	1.723875	-0.662314	-0.409396	2.204473	1.683431	0.666759	0.042286
	1.436145	-0.265988	-0.509857	2.569943	1.979720	-0.333383	0.144053
	1.520490	-0.235975	-0.411737	1.299063	1.833674	-0.249657	0.089249
	1.999530	-0.078149	-0.221351	2.409293	1.964465	-0.346486	-0.157432
	2.280691	-0.670683	-0.316506	2.406616	1.764487	-0.071464	-0.118155
mean	1.792146	-0.382622	-0.373769	2.177878	1.845155	-0.066846	0.000000

Relative expression = $2^{-\Delta\Delta Cq}$

Amddc	Queens			Workers			
	2-3 days	4-5 days	7 days (adult)	2-3 days	4-5 days	7-8 days	10 days (adults)
	0.302735	1.582619	1.328130	0.216964	0.311341	0.629920	0.971115
	0.369554	1.202459	1.423909	0.168411	0.253539	1.259965	0.904973
	0.348567	1.177702	1.330286	0.406390	0.280549	1.188925	0.940012
	0.250081	1.055663	1.165825	0.188248	0.256234	1.271460	1.115300
	0.205799	1.591826	1.245311	0.188598	0.294331	1.050782	1.085346
mean	0.295347	1.322054	1.298692	0.233722	0.279199	1.080210	1.003349
SE	0.030382	0.111080	0.043616	0.043855	0.011067	0.119234	0.041222

- *Amnat*

Average Cq-values in *Amnat*

Amnat	Queens			Workers			
	2-3 days	4-5 days	7 days (adult)	2-3 days	4-5 days	7-8 days	10 days (adults)
	18.697746	17.127544	15.885488	17.633095	17.695064	16.529233	14.419738
	18.061613	17.202554	15.713332	18.056258	18.075419	16.356506	15.333376
	17.622509	16.886648	15.682977	18.304846	18.172743	16.279730	15.642671
	18.226614	17.193880	15.624176	17.405609	17.865228	16.304309	15.679347
	17.370618	16.418383	14.636882	17.570297	17.873879	16.421381	15.062495
mean	17.995820	16.965802	15.508571	17.794021	17.936466	16.378232	15.227525
SE	0.232436	0.148352	0.222218	0.166812	0.084381	0.044890	0.231025

ΔCq = target Cq - *Amtbpaf* Cq

Amnat	Queens			Workers			
	2-3 days	4-5 days	7 days (adult)	2-3 days	4-5 days	7-8 days	10 days (adults)
	1.739985	0.044358	-1.329223	0.686130	1.165947	-0.066435	-2.305503
	1.274969	0.056154	-1.539204	1.335174	1.331377	-0.547421	-1.451422
	0.821977	-0.199597	-1.513000	-0.068010	1.163806	-0.677346	-1.763382
	1.286604	-0.084722	-1.794524	0.835641	1.396404	-0.573323	-1.737020
	0.951275	-0.537796	-2.464361	1.021049	1.198096	-0.618477	-2.260290
mean	1.214962	-0.144321	-1.728062	0.761997	1.251126	-0.496601	-1.903523

ΔΔCq = target ΔCq - average ΔCq of adult workers

Amnat	Queens			Workers			
	2-3 days	4-5 days	7 days (adult)	2-3 days	4-5 days	7-8 days	10 days (adults)
	3.643508	1.947881	0.574300	2.589653	3.069470	1.837088	-0.401980
	3.178492	1.959677	0.364319	3.238697	3.234900	1.356102	0.452101
	2.725500	1.703926	0.390523	1.835514	3.067329	1.226177	0.140141
	3.190127	1.818801	0.108999	2.739164	3.299927	1.330200	0.166503
	2.854798	1.365727	-0.560838	2.924572	3.101619	1.285047	-0.356767
mean	3.118485	1.759203	0.175461	2.665520	3.154649	1.406923	0.000000

Relative expression = $2^{-\Delta\Delta Cq}$

Amnat	Queens			Workers			
	2-3 days	4-5 days	7 days (adult)	2-3 days	4-5 days	7-8 days	10 days (adults)
	0.080019	0.259197	0.671612	0.166126	0.119124	0.279886	1.321320
	0.110453	0.257086	0.776835	0.105939	0.106218	0.390636	0.730977
	0.151197	0.306950	0.762853	0.280192	0.119300	0.427449	0.907430
	0.109566	0.283456	0.927231	0.149772	0.101537	0.397713	0.891000
	0.138236	0.388039	1.475126	0.131709	0.116498	0.410358	1.280553
mean	0.117894	0.298945	0.922731	0.166747	0.112535	0.381208	1.026256
SE	0.012414	0.024056	0.144060	0.030070	0.003645	0.026091	0.116476

- *Amdat*

Average Cq-values in *Amdat*

Amdat	Queens			Workers			
	2-3 days	4-5 days	7 days (adult)	2-3 days	4-5 days	7-8 days	10 days (adults)
	23.303365	22.766545	21.978837	23.027541	23.251334	22.057595	22.127912
	22.534916	22.249025	22.443141	23.527973	22.676166	22.439624	21.967396
	23.310473	22.265692	22.532470	22.665679	22.749417	22.613040	22.296867
	23.473234	23.112351	22.833962	22.906471	22.404496	22.545858	22.254958
	23.034198	21.837290	21.476586	23.315916	23.148663	22.028557	22.287586
mean	23.131237	22.446180	22.252999	23.088716	22.846015	22.336935	22.186944
SE	0.164883	0.222330	0.237672	0.151737	0.156365	0.123198	0.062659

ΔCq = target Cq - *Amtbpaf* Cq

Amdat	Queens			Workers			
	2-3 days	4-5 days	7 days (adult)	2-3 days	4-5 days	7-8 days	10 days (adults)
	6.345605	5.683359	4.764125	6.080576	6.722217	5.461927	5.402671
	5.748272	5.102624	5.190605	6.806889	5.932125	5.535698	5.182598
	6.509941	5.179447	5.336493	4.292823	5.740479	5.655965	4.890815
	6.533224	5.833749	5.415261	6.336503	5.935671	5.668225	4.838592
	6.614855	4.881110	4.375343	6.766668	6.472881	4.988699	4.964801
mean	6.350379	5.336058	5.016365	6.056692	6.160675	5.462103	5.055895

ΔΔCq = target ΔCq - average ΔCq of adult workers

Amdat	Queens			Workers			
	2-3 days	4-5 days	7 days (adult)	2-3 days	4-5 days	7-8 days	10 days (adults)
	1.289709	0.627464	-0.291770	1.024680	1.666322	0.406031	0.346776
	0.692377	0.046729	0.134710	1.750994	0.876229	0.479802	0.126703
	1.454045	0.123551	0.280597	-0.763072	0.684584	0.600069	-0.165080
	1.477329	0.777854	0.359366	1.280608	0.879776	0.612330	-0.217304
	1.558960	-0.174785	-0.680552	1.710773	1.416985	-0.067196	-0.091094
mean	1.294484	0.280162	-0.039530	1.000797	1.104779	0.406207	0.000000

Relative expression = $2^{-\Delta\Delta Cq}$

Amdat	Queens			Workers			
	2-3 days	4-5 days	7 days (adult)	2-3 days	4-5 days	7-8 days	10 days (adults)
	0.409033	0.647313	1.224141	0.491519	0.315056	0.754697	0.786339
	0.618834	0.968129	0.910853	0.297097	0.544789	0.717076	0.915923
	0.364997	0.917925	0.823250	1.697100	0.622185	0.659722	1.121229
	0.359153	0.583234	0.779507	0.411622	0.543452	0.654139	1.162559
	0.339396	1.128796	1.602753	0.305496	0.374494	1.047679	1.065178
mean	0.418283	0.849080	1.068101	0.640567	0.479995	0.766663	1.010245
SE	0.051413	0.102112	0.154623	0.266565	0.057783	0.072688	0.069844

- *Amdop1*

Average Cq-values in *Amdop1*

Amdop1	Queens			Workers			
	2-3 days	4-5 days	7 days (adult)	2-3 days	4-5 days	7-8 days	10 days (adults)
	21.646459	20.050498	20.157779	20.240080	19.288068	18.523771	18.945413
	20.767790	20.056661	20.294552	20.735382	20.063332	19.008366	19.255942
	20.292997	19.433636	20.117316	20.232450	19.667037	18.600685	19.747768
	21.038734	20.085283	20.471990	19.998725	19.646584	18.526054	19.987849
	21.092193	19.601124	20.181246	20.249939	19.878836	18.680842	19.722838
mean	20.967635	19.845440	20.244577	20.291315	19.708772	18.667943	19.531962
SE	0.220958	0.136649	0.064013	0.120542	0.129853	0.089860	0.188556

ΔCq = target Cq - *Amtbpaf* Cq

Amdop1	Queens			Workers			
	2-3 days	4-5 days	7 days (adult)	2-3 days	4-5 days	7-8 days	10 days (adults)
	4.688698	2.967312	2.943068	3.293115	2.758951	1.928102	2.220172
	3.981146	2.910261	3.042017	4.014299	3.319291	2.104439	2.471144
	3.492465	2.347391	2.921339	1.859594	2.658100	1.643609	2.341716
	4.098723	2.806681	3.053289	3.428758	3.177759	1.648421	2.571483
	4.672850	2.644944	3.080003	3.700691	3.203054	1.640984	2.400053
mean	4.186777	2.735318	3.007943	3.259291	3.023431	1.793111	2.400913

$\Delta\Delta$Cq = target ΔCq - average ΔCq of adult workers

Amdop1	Queens			Workers			
	2-3 days	4-5 days	7 days (adult)	2-3 days	4-5 days	7-8 days	10 days (adults)
	2.287785	0.566398	0.542154	0.892202	0.358038	-0.472811	-0.180742
	1.580233	0.509347	0.641103	1.613385	0.918377	-0.296474	0.070230
	1.091552	-0.053523	0.520425	-0.541319	0.257186	-0.757304	-0.059198
	1.697810	0.405768	0.652376	1.027844	0.776846	-0.752492	0.170569
	2.271936	0.244031	0.679090	1.299778	0.802140	-0.759929	-0.000860
mean	1.785863	0.334404	0.607030	0.858378	0.622517	-0.607802	0.000000

Relative expression = $2^{-\Delta\Delta Cq}$

Amdop1	Queens			Workers			
	2-3 days	4-5 days	7 days (adult)	2-3 days	4-5 days	7-8 days	10 days (adults)
	0.204790	0.675301	0.686745	0.538791	0.780225	1.387811	1.133467
	0.334428	0.702540	0.641222	0.326831	0.529104	1.228139	0.952486
	0.469256	1.037796	0.697166	1.455303	0.836718	1.690329	1.041886
	0.308254	0.754835	0.636232	0.490442	0.583641	1.684701	0.888492
	0.207052	0.844383	0.624559	0.406189	0.573498	1.693407	1.000596
mean	0.304756	0.802971	0.657185	0.643511	0.660637	1.536878	1.003385
SE	0.048730	0.065407	0.014544	0.206157	0.061696	0.096809	0.041354

- *Amdop2*

Average Cq-values in *Amdop2*

Amdop2	Queens			Workers			
	2-3 days	4-5 days	7 days (adult)	2-3 days	4-5 days	7-8 days	10 days (adults)
	22.604912	22.300777	21.300718	21.770799	21.321608	21.271510	20.461421
	21.898573	22.609147	21.480010	22.077400	21.933820	21.338663	20.579963
	21.381219	22.246500	21.658053	22.241033	21.931888	21.203874	21.255596
	22.050347	22.434034	21.411561	21.546986	21.372208	21.336898	21.540056
	22.073471	22.396244	21.455399	21.982345	21.492701	21.519667	21.391427
mean	22.001704	22.397340	21.461148	21.923713	21.610445	21.334122	21.045693
SE	0.195833	0.062565	0.058033	0.121049	0.134526	0.052602	0.219803

ΔCq = target Cq - *Amtbpaf* Cq

Amdop2	Queens			Workers			
	2-3 days	4-5 days	7 days (adult)	2-3 days	4-5 days	7-8 days	10 days (adults)
	5.647152	5.217591	4.086007	4.823834	4.792491	4.675842	3.736180
	5.111929	5.462747	4.227475	5.356317	5.189779	4.434736	3.795165
	4.580687	5.160255	4.462076	3.868178	4.922951	4.246798	3.849543
	5.110336	5.155432	3.992860	4.977018	4.903383	4.459265	4.123690
	5.654127	5.440065	4.354157	5.433097	4.816919	4.479809	4.068642
mean	5.220846	5.287218	4.224515	4.891689	4.925104	4.459290	3.914644

ΔΔCq = target ΔCq - average ΔCq of adult workers

Amdop2	Queens			Workers			
	2-3 days	4-5 days	7 days (adult)	2-3 days	4-5 days	7-8 days	10 days (adults)
	1.732508	1.302947	0.171363	0.909190	0.877847	0.761198	-0.178464
	1.197285	1.548103	0.312831	1.441673	1.275135	0.520092	-0.119479
	0.666042	1.245611	0.547432	-0.046466	1.008307	0.332154	-0.065101
	1.195692	1.240788	0.078216	1.062374	0.988739	0.544621	0.209046
	1.739483	1.525421	0.439513	1.518453	0.902275	0.565165	0.153998
mean	1.306202	1.372574	0.309871	0.977045	1.010460	0.544646	0.000000

Relative expression = $2^{-\Delta\Delta Cq}$

Amdop2	Queens			Workers			
	2-3 days	4-5 days	7 days (adult)	2-3 days	4-5 days	7-8 days	10 days (adults)
	0.300928	0.405298	0.888003	0.532484	0.544179	0.590006	1.131678
	0.436095	0.341959	0.805061	0.368140	0.413187	0.697327	1.086343
	0.630233	0.421729	0.684237	1.032732	0.497129	0.794350	1.046158
	0.436577	0.423142	0.947228	0.478843	0.503918	0.685571	0.865109
	0.299477	0.347378	0.737384	0.349060	0.535042	0.675878	0.898756
mean	0.420662	0.387901	0.812383	0.552252	0.498691	0.688626	1.005609
SE	0.060594	0.017947	0.047959	0.124865	0.023164	0.032520	0.052542

- *Amdop3*

Average Cq-values in *Amdop3*

Amdop3	Queens			Workers			
	2-3 days	4-5 days	7 days (adult)	2-3 days	4-5 days	7-8 days	10 days (adults)
	23.058607	22.371497	21.948652	22.507428	21.688487	21.565043	21.500362
	22.579409	22.558846	22.090154	23.114331	22.290859	21.788801	22.178711
	22.229711	22.104076	22.225590	22.718873	22.120280	21.509317	22.512513
	22.854217	22.770985	22.070964	22.325901	22.104351	21.536234	22.375090
	22.592025	22.074405	21.753669	22.622777	22.198705	21.743920	22.423922
mean	22.662794	22.375962	22.017806	22.657862	22.080537	21.628663	22.198120
SE	0.140149	0.133111	0.079305	0.131557	0.103453	0.057342	0.182810

ΔCq = target Cq - *Amtbpaf* Cq

Amdop3	Queens			Workers			
	2-3 days	4-5 days	7 days (adult)	2-3 days	4-5 days	7-8 days	10 days (adults)
	6.100847	5.288311	4.733941	5.560463	5.159370	4.969374	4.775121
	5.792765	5.412446	4.837618	6.393247	5.546818	4.884874	5.393913
	5.429179	5.017831	5.029613	4.346017	5.111343	4.552242	5.106461
	5.914207	5.492383	4.652263	5.755933	5.635527	4.658601	4.958724
	6.172681	5.118225	4.652426	6.073529	5.522922	4.704062	5.101138
mean	5.881936	5.265839	4.781172	5.625838	5.395196	4.753831	5.067071

ΔΔCq = target ΔCq - average ΔCq of adult workers

Amdop3	Queens			Workers			
	2-3 days	4-5 days	7 days (adult)	2-3 days	4-5 days	7-8 days	10 days (adults)
	1.033775	0.221240	-0.333130	0.493391	0.092299	-0.097697	-0.291951
	0.725694	0.345375	-0.229453	1.326176	0.479746	-0.182197	0.326842
	0.362108	-0.049241	-0.037458	-0.721054	0.044272	-0.514830	0.039389
	0.847135	0.425312	-0.414808	0.688862	0.568455	-0.408470	-0.108347
	1.105610	0.051154	-0.414645	1.006457	0.455851	-0.363009	0.034066
mean	0.814865	0.198768	-0.285899	0.558767	0.328125	-0.313240	0.000000

Relative expression = $2^{-\Delta\Delta Cq}$

Amdop3	Queens			Workers			
	2-3 days	4-5 days	7 days (adult)	2-3 days	4-5 days	7-8 days	10 days (adults)
	0.488430	0.857828	1.259744	0.710353	0.938027	1.070064	1.224294
	0.604706	0.787103	1.172391	0.398824	0.717104	1.134610	0.797280
	0.778027	1.034720	1.026304	1.648386	0.969779	1.428825	0.973067
	0.555887	0.744678	1.333121	0.620343	0.674338	1.327277	1.077993
	0.464706	0.965164	1.332971	0.497767	0.729080	1.286105	0.976664
mean	0.578351	0.877899	1.224906	0.775135	0.805666	1.249376	1.009859
SE	0.055709	0.054131	0.057794	0.224640	0.061404	0.065170	0.070096

- *Amgpcr19*

Average Cq-values in *Amgpcr19*

Amgpcr19	Queens			Workers			
	2-3 days	4-5 days	7 days (adult)	2-3 days	4-5 days	7-8 days	10 days (adults)
	19.617665	18.915418	18.961719	18.814166	18.628212	18.822820	18.895117
	18.697777	18.808003	18.903625	18.789111	19.104439	19.219793	19.208289
	18.158202	18.652334	19.092135	19.107614	19.111569	19.186907	19.491990
	18.856652	19.073911	18.575865	18.362910	18.684236	19.150325	19.148296
	18.960611	19.052212	18.590254	18.721260	18.831416	19.410469	19.530429
mean	18.858181	18.900376	18.824720	18.759012	18.871974	19.158063	19.254824
SE	0.234842	0.078566	0.103298	0.119120	0.101921	0.095079	0.117283

ΔCq = target Cq - *Amtbpaf* Cq

Amgpcr19	Queens			Workers			
	2-3 days	4-5 days	7 days (adult)	2-3 days	4-5 days	7-8 days	10 days (adults)
	2.659904	1.832232	1.747008	1.867201	2.099095	2.227152	2.169876
	1.911133	1.661603	1.651089	2.068028	2.360398	2.315866	2.423491
	1.357670	1.566089	1.896158	0.734759	2.102632	2.229831	2.085937
	1.916641	1.795309	1.157164	1.792943	2.215411	2.272693	1.731930
	2.541267	2.096033	1.489012	2.172012	2.155633	2.370611	2.207644
mean	2.077323	1.790253	1.588086	1.726988	2.186634	2.283230	2.123776

ΔΔCq = target ΔCq - average ΔCq of adult workers

Amgpcr19	Queens			Workers			
	2-3 days	4-5 days	7 days (adult)	2-3 days	4-5 days	7-8 days	10 days (adults)
	0.536129	-0.291544	-0.376768	-0.256575	-0.024681	0.103376	0.046101
	-0.212643	-0.462173	-0.472687	-0.055748	0.236622	0.192090	0.299715
	-0.766106	-0.557687	-0.227617	-1.389017	-0.021144	0.106055	-0.037838
	-0.207134	-0.328466	-0.966612	-0.330833	0.091636	0.148917	-0.391846
	0.417492	-0.027743	-0.634764	0.048236	0.031857	0.246835	0.083868
mean	-0.046453	-0.333523	-0.535690	-0.396787	0.062858	0.159455	0.000000

Relative expression = $2^{-\Delta\Delta Cq}$

Amgpcr19	Queens			Workers			
	2-3 days	4-5 days	7 days (adult)	2-3 days	4-5 days	7-8 days	10 days (adults)
	0.689619	1.223949	1.298430	1.194639	1.017255	0.930852	0.968551
	1.158809	1.377615	1.387691	1.039398	0.848730	0.875337	0.812413
	1.700673	1.471907	1.170900	2.619002	1.014764	0.929125	1.026575
	1.154393	1.255678	1.954245	1.257739	0.938458	0.901927	1.312071
	0.748725	1.019416	1.552684	0.967118	0.978160	0.842743	0.943524
mean	1.090444	1.269713	1.472790	1.415579	0.959473	0.895997	1.012627
SE	0.181463	0.076637	0.135455	0.305335	0.031185	0.016749	0.082663
